# The effect of green tea extract on the sperm parameters and histological changes of testis in rats exposed to para-nonylphenol

**DOI:** 10.18502/ijrm.v17i10.5290

**Published:** 2019-11-07

**Authors:** Parisa Azizi M.Sc., Malek Soleimani Mehranjani Ph.D.

**Affiliations:** Department of Biology, Faculty of Sciences, Arak University, Arak, Iran.

**Keywords:** Green tea extract, Para-nonylphenol, Sperm, Testis, Rat.

## Abstract

**Background:**

Para-nonylphenol (p-NP), an environmental contaminant, can generate free radicals that disturbs the reproductive properties. Green tea extract (GTE) is an antioxidant which may prevent the adverse effects of free radicals. **Objective:** The aim was to investigate the effect of GTE on sperm parameters and testis tissue in p-NP-treated rats.

**Materials and Methods:**

24 adult male Wistar rats (215 ± 20 gr) were randomly divided into four groups (n = 6/each) – including control, p-NP (200 mg/kg/day), GTE (200 mg/kg/day), and p-NP + GTE – and orally treated for 56 days. The right testes and left caudal epididymis were used to evaluate selected parameters. In addition, the concentration of serum malondialdehyde was calculated.

**Results:**

A significant decrease in the sperm number, motility, viability and morphology (p < 0.001) was observed in the rats treated with p-NP compared to the control ones. The diameter of seminiferous tubules (p < 0.001), thickness of germinal epithelium (p = 0.018), total volume of testis (p = 0.009), volume of seminiferous tubules (p < 0.001), and testis weight (p = 0.017) decreased in the p-NP group in contrast with the other groups. Moreover, a significant increase of the malondialdehyde concentration was seen in the p-NP group when compared with the controls (p = 0.043). The majority of adverse effects of p-NP could be recovered following the administration of GTE.

**Conclusion:**

It seems GTE can be used as a potent antioxidant in the case of p-NP toxication.

## 1. Introduction

Spermatogenesis is a highly intricate developmental process with the goal of generating mature spermatozoa. This process is characterized by well-defined progressive stages of proliferative expansion, meiosis, as well as cytodifferentiation. There are several factors such as toxins, chemotherapeutic drugs, and environmental pollutants which can adversely impact spermatogenesis (1). There exists an increasing concern over the effects of environmental pollutants on the male reproductive system of the humans and animals. These substances can enter the human body through food, water, air, and skin contact (2). Nonylphenols (NP) are regarded as one of the alkylphenols that are relatively persistent and can easily accumulate in living organisms. They are widely used in the preparation of lubricating oil additives, plasticizers, and surface active agents. Nonylphenols (NP) have also been used as heat stabilizers for poly *vinyl chloride* (*PVC*) and as substantial materials in food processing and packaging industry (3). NP can decrease the activity of scavenging enzymes, including superoxide dismutase and glutathione peroxidase in the testis which in turn leads to oxidative damage (4). It also induces p53-Bcl${}_{2}$/Bax and Fas/FasL signaling pathway in the germ cells, causing apoptosis (5).

Moreover, a recent study shows that phenolic compounds as potential antioxidants can protect cells against toxic chemicals. Green tea (*Camellia sinensis*) is among the most commonly used drinks in the world (6). It contains many ingredients including catechins, caffeine, theanine, and vitamins (7). Tea polyphenols in green tea possess lots of catechins including catechin, epicatechin (EC), epicatechin-3-gallate (ECG), epigallocatechin (EGC), and epigallocatechin-3-gallate (EGCG) (8). Although the precise mechanisms of green tea polyphenols functions have not been specified, More recent studies *have* shown that *polyphenols* (*green tea*) have the capacity to scavenge the toxic reactive oxygen species (ROS) and to trap hydroxyl and peroxyl radicals (9).

The main objective of the present research was to investigate the toxic effects of p-NP on the reproductive system of male albino rats as well as studying the impact of green tea extract (GTE) on the toxicity induced by p-NP.

## 2. Materials and Methods

### Animals and treatments

In the present study, 24 adult male Wistar rats with an average weight of 215 ± 20 gr were purchased from the Iran Pasteur Institute and kept in Arak University animal house under controlled environmental conditions (22 ± 2°C and 12 hr light/dark). The rats were randomly divided into four groups (n = 6/each): Control group (2 ml normal saline), p-NP (200 mg/kg/day; Merck, Germany), GTE (200 mg/kg/day, Institute of Medicinal Plants, Iran), and p-NP + GTE. Since the duration of spermatogenesis in Wistar rats has been demonstrated to be 52 days (10), the oral treatment was carried out through gavage for 8 wk. Corn oil (11) was used as a carrier for p-NP and water (8) was used as a carrier for GTE.

### Surgical procedure

At the end of the treatments, the rats were weighed and killed following ether anesthesia. The right testes and left caudal epididymis were taken out. Then, the *epididymis* of *each* rat was *cut* and placed *in *10 ml Ham's F10 medium to evaluate sperm parameters. Blood samples were also taken from the animals' hearts to measure serum levels malondialdehyde (MDA) levels. Likewise, the testes weight was individually recorded. To obtain the volume of testis, the immersion method (12) was used and then fixed in a modified Davidson's fluid(mDF) for 1 wk (13).

### Estimation of sperm number, motility, viability, and morphology

To specify the sperm count, left caudal epididymis was used. The dissected epididymis was placed in 10 ml Ham's F10 medium and cut into smaller segments in order to swim out the sperm into the medium. After 10 minutes, 1 ml suspension was diluted with 9 ml of formaldehyde solution. The Neubauer method was used to measure the sperm count, expressed as million per ml (14).

The sperm motility was determined using the conventional methods proposed by the World Health Organization (WHO) guidelines (14). Sperm samples (10 μl) were transferred into a semen analysis chamber and immediately assessed. Within each measurement, a minimum of five microscopic fields were evaluated to estimate the motility of 200 sperms per sample. The percentage of sperm motility was analyzed according to the following motion patterns: progressively motile sperm (PMS), non-PMS (NPMS), and non-motile sperm (NMS).

To determine the sperm viability, Eosin-Nigrosin staining was used according to the WHO protocol (14). First, the solutions of eosin (1%) and Nigrosin (10%) were separately provided in distilled water. Then, 1 volume of sperm solution was mixed with 2 volumes of 1% eosin solution. After 30 seconds, an equal volume of Nigrosin solution was added to this mixture. Next, thin smears were also prepared and observed using a light microscope at 1000× magnification. In this method, viable sperms remained colorless while nonviable ones stained red. Smears were prepared and stained with Eosin-Nigrosin in order to analyze sperm morphology. One hundred sperms were counted for each animal, and the abnormal shaped ones were recorded.

### Estimation of sperm chromatin quality 

To test the integrity of sperm DNA, thin smears were prepared from the sperm solution and allowed to air-dry using two different methods, including acridine orange (AO) test and aniline blue (AB) staining (15). Briefly, the dried smears were placed for 14 hrs in methanol/acetic acid (3:1) at 4°C and stained with AO solution (0.19% in phosphate-citrate buffer, pH = 2.5) for 10 min (15). After washing and drying and also immediately after AO staining, the slides were examined using a fluorescent microscope at 1000× magnification. On each slide, an average of 100 spermatozoa was counted. Three types of staining patterns were considered in the sperm head; green spermatozoa (double-stranded DNA), yellow, and red spermatozoa (single-stranded DNA).

Also, the air-dried smears were placed in 4% formalin solution for 5 min, rinsed in distilled water, and stained in 5% AB in 4% acetic acid (pH 3.5) solution for 5 min. The slides were washed in distilled water, stained in 0.5% eosin for 1 min and allowed to air-dry (15). The slides were then examined at 1000× magnification using a light microscope. Immature sperms which are characterized by the presence of nuclear histone proteins were stained dark blue, whereas mature sperms with protamine were stained red-pink.

### Stereological and histological study

Isotropic Uniform Random (IUR) sections of testis tissue were prepared using the orientator method (4). For this purpose, the testis tissue was randomly placed on the φ clock each half of which was divided into nine equal parts. After that, numbers were randomly selected within the range of 0 and 9 and a proper cut was made along the selected number. Then, one piece was placed vertically on the θ-clock each half of which was divided into nine unequal parts. Next, the second piece was selected randomly and a parallel cut was made along the selected number. The other piece was also placed parallel to the θ-clock along its cut surface on the 0–0 axis, and a random number was selected and another cut was made along the selected number. In order to calculate the shrinkage rate, the round segments**from the**testicular tissue sections**were prepared with a trocar. The vertical diameters of round segments were measured and their mean radius was estimated and considered as the pre fixing radius (r. before). After processing and embedding the tissue, 5 μm-thick sections were cut using a microtome and stained with Heidenhains AZAN staining method.

### Estimating the shrinkage and the total volume of the testis

After processing, sectioning, and staining the tissue, the trocar radiuses were again measured and the obtained mean radius was considered as the post-fixing radius (r. after). Subsequently, the amount of shrinkage in each testis was calculated using the following equation (16): 

 Shrinkage =r after 2r befor 232.

Finally, to measure the total volume of the testis, the amount of shrinkage was subtracted from the volume estimated by the immersion method.

### Estimating the volume of seminiferous tubules 

To estimate the volume of seminiferous tubules, the systematic random sampling method was used. Doing so, an average of five to seven random fields per each 5 µm-thick sections were evaluated by placing the point probe on each field. Then, the total points superimposed on the whole field $\left(\sum_{i=1}^{n}Ptotal\right)$, along with the total points superimposed on the seminiferous tubules $\left(\sum_{i=1}^{n}Ptubules\right)$ were counted. The volume density of each fraction was calculated by the following equation:

$$V_{v}=\frac{\sum_{i=1}^{n}Ptubules}{\sum_{i=1}^{n}Ptotal}.$$

Eventually, the total volume of each fraction was obtained by multiplying the volume density (Vv) by the total volume of the testis (4).

### Estimating the diameter of seminiferous tubules and the height of the germinal epithelium

5 μm-thick sections were used to evaluate the diameter of seminiferous tubules and the height of the germinal epithelium. For this purpose, a number of sections were randomly selected and photographed separately from random scopes. Then these parameters were measured by Motic *Image 2000 *software. The mean of each parameter (µm) was determined for each testis (17).

### Measurement of serum MDA level

MDA serum concentration, an important indicator of lipid peroxidation, was measured according to the method described by Sadeghzadeh and colleagues (16). For this purpose, 1 mL of Trichloroacetic acid (TCA), thiobarbituric acid (TBA) and *hydrochloric acid (HCl*) solution were mixed with 500 µl of sample. The samples were then placed in boiling water for 15 min. After that, it was cooled and centrifuged for 10 min. Next, the supernatant was removed and the absorbance was measured at 535 nm. The extinction coefficient was used to calculate the concentration of MDA, which was 1.56 × 10${}^{5}$ mol${}^{-1}$cm${}^{-1}$ expressed as nmol/mL.

### Ethical consideration

All of the experimental procedures were approved by the local ethical committee of the Arak University.

### Statistical analysis 

The results were analyzed by one-way analysis of variance (ANOVA) and Tukey's test, using the -Statistical Package for the Social Sciences (SPSS software), version 16.0, SPSS Inc, Chicago, Illinois, USA, and the means were considered significantly different at p < 0.05.

## 3. Results

### Sperm motility

The mean percentage of PMS (p < 0.001) decreased significantly and the percentage of NPMS (p < 0.001) and NMS (p = 0.001) increased significantly in the p-NP group compared to the control, while the mean number of PMS and NMS was similar in the p-NP + GTE when compared to the control group (Table I).

### Sperm viability

A significant decrease in the mean percentage of viable sperms (p < 0.001) was found in the p-NP group compared to the control group but it increased significantly to the control level in the p-NP + GTE group (Table I).

### Sperm number

As revealed in Table I, the mean sperm number (p < 0.001) reduced significantly in the p-NP group compared to the control, while no significant difference was found when comparing the mean sperm number in the p-NP and the p-NP + GTE groups (Table I).

### Sperm morphology

**Figure 1:**
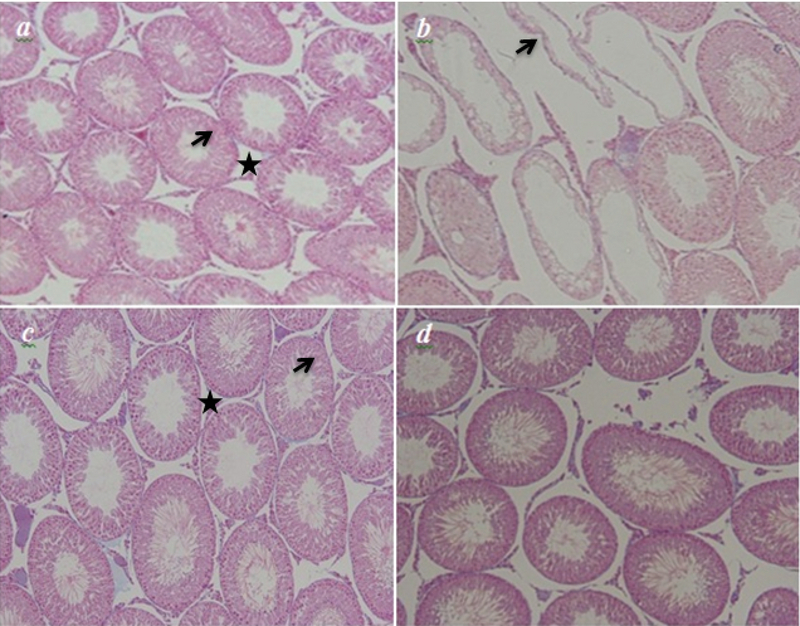
Investigating sperm abnormalities in p-NP-treated rats using Eosin-Nigrosin Staining (Magnification: 1000×). (a) Banana head, (b) Coiled tail, (c) Pin head.

The mean percentage of morphologically normal sperms (p < 0.001) showed a significant decrease in the p-NP group compared to the control (Table I), while it increased significantly to the control level in the p-NP + GTE group. A number of sperm morphological abnormalities induced in p-NP-treated animals are shown in Figure 1.

### Sperm chromatin quality

The sperm DNA integrity and histone-protamine replacement showed no significant difference in the p-NP group compared to the control.

### Histopathological findings

**Figure 2:**
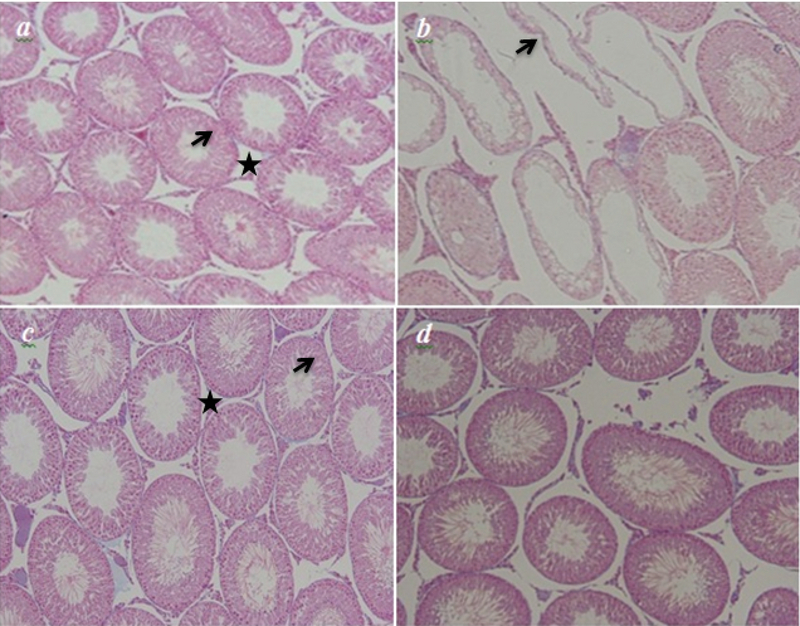
Histopathological analysis of the p-NP- and GTE-treated rats using the Light microscopic study. (a) and (c) the control and the GTE groups manifesting a normal feature of the seminiferous epithelium (arrow) and interstitial tissue (star). (b) the p-NP group revealing a markable shrinkage of empty seminiferous tubules (arrow). (d) the p-NP + GTE group – the undesired changes are ameliorated toward the normal structure (Magnification: ×100).

Light microscopic study of the sections obtained from the rats in the control and GTE groups showed normal arrangements of testis tissue and the germinal epithelium (Figure 2a and 2c). In the p-NP group, irregular and vacuolated germinal epithelium with a reduction in height was observed in the structure of the testis (Figure 2b), but these changes restored toward the normal structure in the p-NP + GTE group (Figure 2d).

### Diameter of seminiferous tubules and height of germinal epithelium

A highly significant reduction was seen in the mean diameter of seminiferous tubules and the height of the germinal epithelium in the p-NP group compared to the control group. Moreover, the levels of the afore-mentioned parameters were significantly higher in the p-NP + GTE group (Table II).

### Total volume of testis and seminiferous tubules

The mean total volume of the testis and the volume of the seminiferous tubules significantly decreased in the p-NP group when compared to the control, while a significant increase to the control level in the above parameters was observed in the p-NP + GTE group (Table II).

### Body and testis weight 

A significant reduction in the mean testis weight in the p-NP group was found when compared to the control group (p = 0.017), while it increased significantly to the control level in the p-NP + GTE group. No significant difference was observed in the body weight in any of the groups (p = 0.088) (Table III).

### MDA concentration

**Figure 3:**
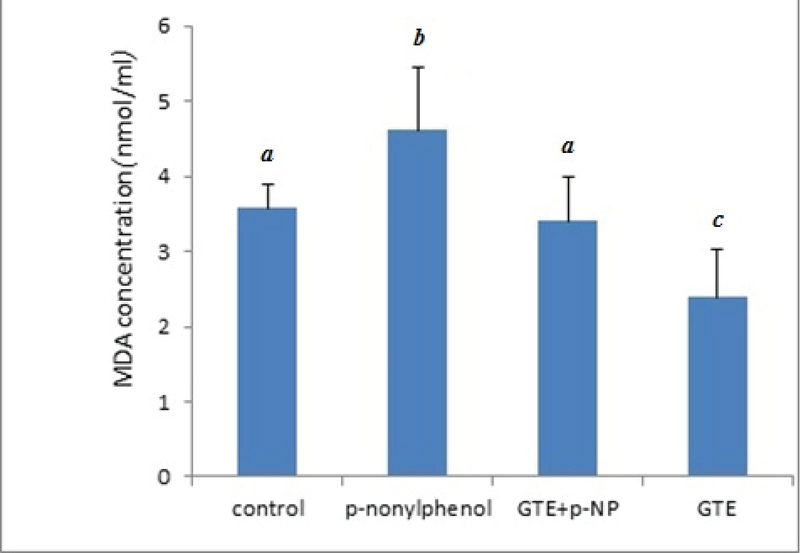
Comparing the MDA concentration (nm/ml) in different groups of rats, 56 days after treatment with p-NP and GTE. Values are means ± SD. Means with different code letter in a column are significantly different (one-way ANOVA and Tukey's test, p < 0.05).

The concentration of serum MDA was significantly higher in the p-NP group compared to the control group (p= 0.043), while it reduced significantly to the control level in the p-NP + GTE group (Figure 3).

**Table 1:** Comparing the sperm motility, viability, number, and morphological normality in different groups of rats, 56 days after treatment with p-NP and GTE


**Groups **	**Control**	**P-NP**	**GTE**	**P-NP + GTE**	**P-value**
PMS%	73.82 ± 3.55${}^{a}$	60.61 ± 4.09${}^{b}$	81.74 ± 2.46${}^{c}$	71.15 ± 6.16${}^{a}$	< 0.001
NPMS%	15.29 ± 2.49${}^{ac}$	22.69 ± 4.44${}^{b}$	10.52 ± 1.76${}^{a}$	17.32 ± 3.60${}^{c}$	< 0.001
NMS%	10.87 ± 1.79${}^{a}$	16.68 ± 2.53${}^{b}$	7.72 ± 1.23${}^{a}$	11.50 ± 5.36${}^{a}$	0.001
SV%	71.59 ± 2.23${}^{a}$	66.34 ± 4.48${}^{b}$	84.65 ± 2.05${}^{c}$	74.63 ± 1.90${}^{a}$	< 0.001
SMN%	88.56 ± 2.69${}^{a}$	82.66 ± 1.57${}^{b}$	89.78 ± 1.58${}^{a}$	88.44 ± 1.54${}^{a}$	< 0.001
SN (×10${}^{6}$)	16.58 ± 2.13${}^{ac}$	11.91 ± 2.03${}^{b}$	18.08 ± 2.31${}^{c}$	14.50 ± 1.92${}^{ab}$	< 0.001
Data presented as mean ± SD. Means with different code letter in a row are significantly different (one-way ANOVA and Tukey's test, p < 0.05)					
P-NP: Para-nonylphenol; GTE: Green tea extract; PMS: Progressively motile sperm; NPMS: Non-progressively motile sperm; NMS: Non-motile sperm; SV: Sperm viability; SMN: Sperm morphological normality; SN: Sperm number					

**Table 2:** Comparing the diameter of the seminiferous tubules, the height of germinal epithelium, the total volume of the testis, and the seminiferous tubules in different groups of rats, 56 days after treatment with p-NP and GTE


**Groups**	**Control**	**p-NP**	**GTE**	**p-NP + GTE**	**P-value**
STD (µm)	281.26 ± 8.09 ${}^{a}$	234.53 ± 21.25 ${}^{b}$	282 ± 7.32 ${}^{a}$	262.53 ± 10.40 ${}^{a}$	< 0.001
GEH (µm)	76.05 ± 2.90${}^{a}$	68.69 ± 3.98${}^{b}$	79.31 ± 4.91${}^{a}$	78.84 ± 3.34${}^{a}$	0.018
TV (mm${}^{3}$)	1056.92 ± 31.88${}^{a}$	998.20 ± 20.54${}^{b}$	1078.91 ± 24.90${}^{a}$	1058.91 ± 33.45${}^{a}$	0.009
STV (mm${}^{3}$)	791.29 ± 52.29${}^{a}$	701.78 ± 23.95${}^{b}$	839.10 ± 35.82${}^{a}$	811.33 ± 36.90${}^{a}$	< 0.001
Data presented as mean ± SD. Means with different code letter in a row are significantly different (one-way ANOVA and Tukey's test, p < 0.05)					
p-NP: Para-nonylphenol; GTE: Green tea extract; STD: Seminiferous tubules diameter; GEH: Germinal epithelium height; TV: Testis volume; STV: Seminiferous tubules volume					

**Table 3:** Comparing the body and testis weights in different groups of rats, 56 days after treatment with p-NP and GTE


**Group**	**Control**	**p-NP**	**GTE**	**p-NP + GTE**	**P-value**
BW before treatment (gr)	217.50 ± 7.34	215.33 ± 10.72	213.33 ± 15.00	214.33 ± 3.32	0.903
BW after treatment (gr)	296.50 ± 19.12	259.16 ± 23.31	283.50 ± 24.38	277.50 ± 28.15	0.088
Testis weight (gr)	1.44 ± 0.04${}^{a}$	1.29 ± 0.10${}^{b}$	1.47 ± 0.06${}^{a}$	1.44 ± 0.03${}^{a}$	0.017
Data presented as mean ± SD. Means with different code letter in a row are significantly different (one-way ANOVA and Tukey's test, p < 0.05)					
BW: Body weight; p-NP: Para-nonylphenol; GTE: Green tea extract					

## 4. Discussion

The results revealed a significant decrease in sperm motility and viability after treatment with p-NP compared with control group which has been confirmed by other studies (5, 18). This is mainly related to the increase in lipid peroxidation in the sperm membrane (18). Sperm membrane possesses a high level of unsaturated fatty acids making spermatozoa susceptible to oxidative stress. Normal levels of ROS generated by spermatozoa are needed for its physiological processes such as sperm capacitation and the acrosome reaction, however, excessive production of ROS results in a reduced-mitochondrial membrane potential, a reduction in energy availability and consequently reduced sperm motility and viability (19).

In this study, a significant reduction in the sperm number was found in the P-NP-treated group which has been also reported previously by other studies (18). This could either be attributed to the reduction in plasma testosterone level (20) or P-NP-induced lipid peroxidation and H${}_{2}$O${}_{2}$ production (21, 22) which can in turn lead to a reduction in spermatogenesis and sperm count. In addition, p-NP reduces cell proliferation and induces apoptosis through the activation of the p53-Bcl${}_{2}$/Bax and Fas/FasL pathway which impairs the testicular development and function (5). The results also showed an increase in the percentage of morphologically abnormal sperms of the p-NP group compared to the control group which is in accordance with the results obtained by Ansoumane Kourouma and colleagues (23). Normal morphology is an essential indicator for the maturity of the sperm population and correlates with fertility (24).

The results of AO and AB staining revealed no significant differences in the p-NP group compared to the control. Although these results are in agreement with the findings reported by Uguz and colleagues (25) regarding the analysis of p-NP on the DNA integrity, we did not find any similar reports on histon-protamine replacement. Perhaps the effect of p-NP on this parameter depends on the dose and duration of treatment. We also reported a significant reduction in the mean diameter of the seminiferous tubules and the germinal epithelium height in the p-NP group compared to the other groups which is in agreement with other studies (4). Reduction in the activity of scavenging enzymes and induction of oxidative stress as a result of p-NP treatment along with its estrogenic property can cause these abnormalities in the testis tissue (4).

In the present study, the volume of seminiferous tubules reduced significantly in the p-NP group which is also indicated by the results obtained by Mehranjani and colleagues, who showed that the treatment of animals with p-NP lead to atrophied seminiferous tubules (4). Our results revealed a significant reduction in the total volume of the testis after treatment with p-NP. Since the seminiferous tubules are the main components of the testis, so it can be concluded that a reduction in the volume of the seminiferous tubules could be the most important reason explaining the reduction in the volume of the testis. MDA as a lipid peroxidation marker is usually used to assess oxidative stress. An increase in the MDA levels could be a direct reflection of oxidative injuries (16) which can induce tissue damage through lipid peroxidation (26). Our results showed a significant increase in the MDA serum level in the p-NP group compared to the control group, which is in consistent with the other studies (27).

In the present study, the body weight did not differ significantly after treatment with p-NP, indicating that there was no significant change in basal metabolism rate. Although organ weight loss is not always associated with the body weight reduction, it is a basic benchmark for the toxicological studies (11). In this regard, the significant reduction found in the testes weight in this study may be due to the abnormal changes in the seminiferous tubules and germinal cells (4, 28).

In this investigation, we demonstrated that co-administration of p-NP and GTE could compensate the adverse effects of p-NP such as the increase in the serum MDA level, reduction in the sperm motility, viability, morphology, testis weight, seminiferous tubule diameter, germinal epithelium height, volume of the testis and seminiferous tubule, which is probably due to its antioxidant properties that ameliorates the activity of sperm antioxidant defense system including superoxide dismutase, glutathione peroxidase, and catalase (29). GTE also increases the level of scavenging enzymes, including catalase which leads to decreased MDA levels (30).

## 5. Conclusion

We conclude that GTE is able to ameliorate the adverse effects of p-NP on the structure of the testis and sperm parameters in adult rats and therefore the consumption of GTE is suggested as a potential antioxidant in the case of p-NP exposure.

##  Conflict of Interest

The authors would like to declare that there is no conflict of interest.
